# Latin America: A Development Pole for Phenomics

**DOI:** 10.3389/fpls.2016.01729

**Published:** 2016-12-06

**Authors:** Anyela V. Camargo, Gustavo A. Lobos

**Affiliations:** ^1^The National Plant Phenomics Centre, Institute of Biological, Environmental and Rural Sciences, Aberystwyth UniversityAberystwyth, UK; ^2^Facultad de Ciencias Agrarias, Plant Breeding and Phenomic Center, PIEI Adaptación de la Agricultura al Cambio Climático (A2C2), Universidad de TalcaTalca, Chile

**Keywords:** LAC, climate change, genomic, phenotyping, plant breeding, LatPPN

## Abstract

Latin America and the Caribbean (LAC) has long been associated with the production and export of a diverse range of agricultural commodities. Due to its strategic geographic location, which encompasses a wide range of climates, it is possible to produce almost any crop. The climate diversity in LAC is a major factor in its agricultural potential but this also means climate change represents a real threat to the region. Therefore, LAC farming must prepare and quickly adapt to an environment that is likely to feature long periods of drought, excessive rainfall and extreme temperatures. With the aim of moving toward a more resilient agriculture, LAC scientists have created the Latin American Plant Phenomics Network (LatPPN) which focuses on LAC's economically important crops. LatPPN's key strategies to achieve its main goal are: (1) training of LAC members on plant phenomics and phenotyping, (2) establish international and multidisciplinary collaborations, (3) develop standards for data exchange and research protocols, (4) share equipment and infrastructure, (5) disseminate data and research results, (6) identify funding opportunities and (7) develop strategies to guarantee LatPPN's relevance and sustainability across time. Despite the challenges ahead, LatPPN represents a big step forward toward the consolidation of a common mind-set in the field of plant phenotyping and phenomics in LAC.

## Why phenomics is key to face climate change and food security?

In the past decades, climatic variations related to El Niño or La Niña phenomena have brought serious challenges to the agricultural sector in LAC. While drought is the main threat to food production associated to La Niña, El Niño can cause heavy rains, flooding or extremely hot or cold weather (Allen and Ingram, 2002). In the last 150 years, earth's temperature increased at a rate of 0.045°C per decade, with almost four-fold (0.177°C) in the last 25 years (IPCC, 2007), and will continue to raise by another 1.1–6.4°C over the next century (Jin et al., 2011). This increase in temperature can lead to several agricultural associated problems such as yield reduction as a results of droughts, and the emergence and spreading of plant diseases and pests (FAO, 2016). Therefore, a better use of plant genetic resources and plant breeding (Borrás and Slafer, 2008), are key to tackling the imminent impact of climate change in food security. Further, a multidisciplinary approach that includes disciplines such as omics technologies (e.g., genomics, phenomics, proteomics, and metabolomics), plant physiology, eco-physiology, plant pathology and entomology, and soil science will be critical to increase crop resilience to climate change (Reynolds et al., 2016). Undoubtedly, public and private breeding programs have the challenge of producing stress tolerant cultivars whose yield potential and quality are also high. In order to increase the chances of producing desirable cultivars, breeders make a high number of crosses (e.g., Chilean wheat breeding programs generate ~800 crosses per year) and screen them under a limited number of environmental conditions (Araus and Cairns, 2014). Line crossing is a common experimental design for mapping quantitative trait loci (QTLs) in plant breeding. Crosses are initiated from at least two inbred lines, such as backcrosses, F2, and more derived generations (Xie et al., 1998). To increase the statistical inference space of the estimated QTL variance and ensure that polymorphic alleles are present in the parental gene pool, a sufficient number of parents must be sampled (Muranty, 1996). The number of traits measured per plot is normally limited to the size of the population. Increasing the number of traits to be measured requires additional time, resources and the use of skilled labor (Kipp et al., 2014). This represents a limitation toward the understanding of the interaction genotype × environment (G × E) (Furbank and Tester, 2011; Yang et al., 2014; Großkinsky et al., 2015; Rahaman et al., 2015).

Although, genome sequencing has become relatively fast, cheap, and easy to produce, plant phenomics still lags behind. This unbalance has become a bottleneck in the understanding of G × E and it also limits the possibility of carrying out tests under field conditions (Lobos and Hancock, 2015). Therefore, there is a need to incorporate the evaluation of multiple morpho-physiological and physico-chemical traits at the high-throughput level to be able to understand for example pleiotropy or genomic variants that gave rise to a particular phenotype (Houle et al., 2010; Fahlgren et al., 2015).

Due to the cost of high-throughput plant phenotyping, several international phenotyping networks have been established with the idea of joining efforts and produce research with impact. Some of the most prominent networks are: the European Plant Phenotyping Network (EPPN), Food and Agriculture COST Action FA1306, the International Plant Phenotyping Network (IPPN), the Australian Phenomics Network (APN), the German Plant Phenotyping Network (DPPN) and the U.K. Plant Phenomics Network (UKPPN). In Asia, the 1st Asia-Pacific Plant Phenotyping will be held in Beijing, China in October 2016 and the 3rd International Plant Phenotyping Symposium was held in Chennai, India in 2014. More recently in North America, the United States of America recently launched the North American Plant Phenotyping Network (NAPPN).

## Does latin america and the caribbean need to worry about phenomic development?

Latin America is a region that includes Mexico, the Spanish/Portuguese speaking countries in Central America and the whole of South America, as well as the Caribbean (Latin America and the Caribbean—LAC). The region is highly heterogeneous in terms of climate, ecosystems, human population distribution, politics, economy and incomes, and cultural traditions. Out of a total of 17 megadiverse countries identified by the World Conservation Monitoring Centre (http://www.unep-wcmc.org), six are in Latin American, namely Brazil, Colombia, Ecuador, Mexico, Peru, and Venezuela. Furthermore, from the eight primary centers of origin and diversity, numbers VII (South Mexican and Central American) and VIII (South America Andes region: Bolivia, Peru, Ecuador; VIIIa The Chilean Center, and VIIIb Brazilian-Paraguayan Center) are based in the region (Vavilov, 1992).

Due to LAC's diverse geography, climate change will impact the region severely. Compared to pre-industrial times, it is estimated that the mean temperature on the region will increase about 4.5°C by the end of the century (Reyer et al., 2015). Temperatures are expected to increase dramatically in the tropics and moderate at the subtropical regions in the north (Mexico) and south (southern Chile, Argentina and Uruguay) (Reyer et al., 2015). Annual precipitations are also likely to increase in Argentina, Uruguay, Brazil, Peru, Ecuador, and Colombia and decrease in the rest of the countries (Reyer et al., 2015). These changes have a direct impact on agricultural crop yields. It's expected that crops such as wheat, soybean and maize will reduce its yield potential, while others such as rice and sugar cane will increase it (Fernandes et al., 2012; Marin et al., 2012).

The economic development of the regions where plant phenotyping and phenomics have been developed in the last 10 years (high-income countries) is completely different to that of LAC. According to the World Bank, around 37% of the LAC population lives under poverty or extreme poverty (World Bank, 2014), and near 60% of the people living in rural areas is under extreme poverty (RIMISP, 2011). Therefore, besides the climate change effects impacting LAC agriculture, there is also a significant knock-on the region economy, affecting particularly the lower socioeconomic strata (Ortiz, 2012).

Although, LAC countries are wealthier, government efforts are mainly focused on priority areas such as education, health, employability, and infrastructure. Research and innovation in areas such as agriculture has been given a low priority. As a result, most Latin American farmers do not have the resources or the support to effectively adapt to a changing climate that is already showing its negative impact in agriculture (Lobos and Hancock, 2015). Therefore, LAC scientists and private sector must work together to develop strategies aiming at moving toward a more resilient agriculture, and one of them is the use of plant phenomics and phenotyping for breeding.

Phenomics has become a powerful research tool to help breeders to generate cultivars adaptable to more challenging environmental scenarios. In the past decade, phenomics has been focused mainly on breeding of grain crops, but their application in other species of relevance for LAC (e.g., fruit, vegetables, forage and others) is almost absent (Lobos and Hancock, 2015).

The potential of recent advances in phenomics encouraged the Plant Breeding and Phenomic Center (Dr. Gustavo A. Lobos, Universidad de Talca, Talca, Chile) and the National Plant Phenomics Centre (Dr. Anyela Camargo, IBERS, Aberystwyth University, U.K.) to organize the First Latin American Conference on Plant Phenotyping and Phenomics for Plant Breeding (November 30^st^ to December 2^nd^ 2015, Talca, Chile). This event had three main goals: (1) bring to Latin American researchers and students, international keynote speakers and plant breeding companies from around the world, to present their ongoing work on plant phenomics and phenotyping for plant breeding; (2) perform a workshop to train Latin American scientists and postgraduate students in the use of key plant phenotyping tools, the analysis of data and the mapping of traits to the genome; and (3) set up the Latin American Plant Phenomics Network (LatPPN), conceived to facilitate the training on high-throughput phenotyping and pre-breeding methodologies, scientific exchange of young/senior researchers and students, and to improve access to resources and research facilities.

The conference covered a broad range of topics such as pre-breeding and breeding strategies, methods to measure and analyse trait data for plant breeding and the strategies to translate research from the bench to the field. International keynote speakers gave seminal talks and chaired the track of their expertise. Challenges and opportunities were also explored such as the handling of the high amount of data generated through high-throughput phenotyping. Multiple ideas were discussed to deal with every particular challenge. Participants also had the opportunity to attend five workshops that covered aspects such as the use of software and equipment for plant phenotyping (mainly by remote sensing), and data handling and manipulation.

The LatPPN, which is chaired for two years by Chile (Dr. Gustavo A. Lobos) and Colombia (Dr. Anyela Camargo), had it first reunion during the 3^rd^ day of the conference. Representatives from LAC (Argentina, Brazil, Chile, Colombia, Ecuador, Mexico, and Uruguay) and from other countries (Australia, Germany, Saudi Arabia, Spain, U.K., and U.S.A.) got together to discuss what LAC's breeding programs needed to do to become more efficiency in terms of plant phenotyping and phenomics. They also discussed the differences between phenotyping and the more complex concept of phenomics. This discussion helped to define where LAC currently stands (more focused on the phenotyping of few traits and low number of genotypes) and where it needs to be in the future (mostly oriented to the multidimensional approach of phenomics, considering a high number of genotypes assessed). For example, the wheat breeding program of INIA Chile, used to consider a classical approximation where the numbers of traits evaluated increases insofar the number of generation progresses: ~9 traits at F2–F5: susceptibility to *Puccinia triticina, P*. *graminis*, and *P*. *striiformis*, plant height, tillering capacity, type of spike, grain color, type of grain, and black point or other grain defects; ~16 at F6–F8: previous ones plus heading date, grain yield, and some grain characteristics such as test weight, protein and gluten content, sedimentation, and seed hardiness; and ~19 at F9–F10 where less than 5% of the original crosses are evaluated: previous ones plus some other required by millers such as W flour value, falling number, and some bakery aptitudes. Today, using spectrometry and thermography, this breeding program is aimed to predict some of these traits but also to consider other 30 morpho-physiological and physico-chemical characters (some examples covered in next section), screening ~800 genotypes per day.

## Is LAC oriented to phenomics or plant phenotyping?

Due to resources' availability such as equipment, skills and infrastructure, LAC has mainly focused on plant phenotyping. Although phenomics in LAC has not yet had a proper expansion, there are some good examples of institutions focusing on it: (i) The International Maize and Wheat Improvement Center (CIMMYT—Mexico) routinely uses remote sensing and high spec sensor technologies to screen for wheat and maize's responses to biotic and abiotic stresses, among them yield and its components, biomass, senescence (stay-green), water stress, and water use efficiency, canopy cover, photosynthetic capacity and activity (Zaman-Allah et al., 2015). Special emphasis is also put on 3D reconstruction for plant height, spike number and biomass determination; (ii) The Plant Breeding and Phenomic Center (University of Talca—Chile) have focused its efforts on the prediction of physiological traits by spectrometry and thermography (e.g., gas exchange, modulated chlorophyll fluorescence, pigments concentration, stem water potential, hydric and osmotic cell potential, cell membrane stability, lipid peroxidation, proline content, C and O isotopic composition) on several breeding programs (wheat, blueberries, alfalfa, strawberries, and quinoa) oriented to abiotic stresses (salt, water deficit and high temperature) (Garriga et al., 2014; Lobos et al., 2014; Estrada et al., 2015; Hernandez et al., 2015), developing also a software for exploratory analysis of high-resolution spectral reflectance data on plant breeding (Lobos and Poblete-Echeverría, in press).

In terms of phenotyping, most research institutes across the region have done some form of low to medium throughput phenotyping, for example: (i) The International Centre for Tropical Agriculture (CIAT—Colombia) is screening root architecture to identify markers associated to drought stress tolerance in beans and grasses (Villordo-Pineda et al., 2015; Rao et al., 2016); (ii) Embrapa (Brazil) uses traditional phenotyping to screen for root morphology in wheat (Richard et al., 2015); (iii) Universidade Federal de Mato Grosso, Brazil, uses traditional phenotyping tools (e.g., gas exchange measurements) to look for photosynthetic responses of tree species to seasonal variations in hydrology in the Brazilian Cerrado and Pantanal (Dalmagro et al., 2016); (iv) Researchers from Argentina uses conventional phenotyping equipment to investigate the response of seed weight and composition to changes in assimilate supply from leaves, to the incident solar radiation reaching the pods and to the combination of both, changes in assimilate supply from the leaves and incident solar radiation on pods of soybean plants (Bianculli et al., 2016), they are also trying to develop low cost tools in order to make that technology accessible to researches from LAC; (v) The International Potato Center (Peru) have improved the screening of potato breeding lines by spectroscopy (Ayvaz et al., 2016); and (vi) INIA (Uruguay) in collaboration with INIA (Chile) and the Plant Breeding and Phenomic Center (University of Talca—Chile), applied genotyping-by-sequencing to identify single-nucleotide polymorphisms, in the genomes of 384 wheat genotypes that were field tested in Chile under three different water regimes (Lado et al., 2013).

## How will LAC benefit from LatPPN?

The conference served as a platform to showcase LAC capabilities, investigate strengths, and weaknesses, and thereby identify where the challenges lie and what the knowledge and the technological gaps between the region and the rest of the world are.

Given LAC's high heterogeneity in terms of climate, ecosystems and genetic diversity, as well as the differences of each country vulnerability to climate change, it was agreed how important it is for LAC's agri-food chain to take a more proactive role in the development of strategies leading to the selection of crops capable to withstand the impact of climate change.

With the aim of identifying what LatPPN needed to do to strength LAC's plant phenotyping and phenomics research, the panel of participants identified the following key challenges: (i) develop LatPPN's own tailored identity: there is not a common crop but rather a wide diversity of them, from grasses to forest species. As previously mentioned, plant phenotyping and phenomics has been developed almost exclusively on cereal improvement, however LatPPN needs to focus on other breeding programs that are important for particular countries. For example: blueberries for Chile (Chile is the biggest exporter of fresh blueberries in the world, ~90,000 ton during 2015/16), potato for Peru (production was estimated to be 4.5 million tons for 2015), tangerines for Uruguay (production was ~6000 tons in 2014), pineapple for Costa Rica (since 2000, pineapple production has increased by nearly 300%, however production is very inefficient, each plant only produces two fruit over a period of 18–24 months, and requires significant amount fertilizer to do so) and Coffee for Colombia (exports account for ~810,000 ton in 2015) and Brazil (exports account for ~2.6 million tons in 2014). The production of these cash crops will face serious challenges (e.g., post-harvest life, or the incidence of physiological disorders, pests and diseases) in the coming decades due to the sensitivity of them to water shortages and heat stress. In this meeting, it was also highlighted: (ii) training on plant phenomics and phenotyping using strategies that allow the participation of several countries at the same time. We are aiming at finding resources to implement distance-training courses using currently available technologies such as webinars and teleconferences; (iii) learn from experienced researchers and current plant phenotyping and phenomics initiatives. In order to facilitate the interaction between researchers and institution, senior researchers on plant phenotyping and phenomics were invited to participate in the first meeting; (iv) since high-throughput phenotyping requires a broad range of capabilities (e.g., programmers, bioinformaticians, statisticians, biologists, agronomists, geneticists, physiologists), is important to promote interdisciplinary work between researchers; (v) identify the state of art of plant phenotyping and phenomics in LAC. In order to identify strengths, opportunities and weaknesses and develop targeted strategies, key information such as breeding programs, researchers, equipment and infrastructure, regional and local financial sources, and capabilities should be surveyed. All this information should be included on the future LatPPN webpage; (vi) distribute efforts on common goals (e.g., researchers from different countries working on the same species or problem), it will be necessary to standardize measurements and protocols; (vii) sharing of equipment and infrastructure; and (viii) LatPPN visibility and presence. To avoid early disenchantment, LatPPN needs to carry out activities to promote the network (e.g., events, postgraduate grants or proposal calls).

In relation to weaknesses, the lack of a permanent budget to run network activities is one of LatPPN's main concerns. Currently, the Director and Co-Director, the executive committee (Dr. Paulo Hermann from EMBRAPA—Brazil and Dr. Gustavo Pereyra from INTA-CONICET—Argentina), and the representative members (three per country in charge of meet the local demands, thematic promotion, and economic resources leveraging) devote part of their time and resources to consolidate the network. However, they are looking into sources of support within LAC and worldwide. At the country level, there are a number of countries that have access to grants provided by their own governments. At regional level, there are a number of organizations such as PROCISUR and PROCITROPICOS, which provide regular grant support for agricultural research initiatives. At international level, there are several organizations such as FAO (the Food and Agricultural Organization), EU (the European Union), and IBS (the Inter-American Development Bank) who support agricultural research in LAC.

Another weakness is LAC's low publication rate and the lack of accessibility of LAC institutions to main bibliographic databases. According to the World Bank, the number of publications produced by the most important economies in LAC in 2012 was 48,622 from Brazil, 13,112 from Mexico, 8,053 from Argentina, 5,158 from Chile and 4,456 from Colombia. Brazil is the only country whose output is equivalent to high-income countries where phenomics have been developing in the last 10 years; U.S.A. (412,542), Germany (101,074), U.K. (97,332), France (72,555), Spain (53,342), and Australia (47,806) (World Bank, 2012). In term of access to bibliographic databases, most of the institutions in the region have limited or no access to main bibliographic databases such as Scopus and Web of Knowledge. This is serious limitation to the dissemination of the work developed in LAC, especially if we are aiming at improving plant breeding programs through the use of plant phenotyping and phenomics.

Despite the weaknesses, currently there are several international research institutes who are already formally collaborating with LAC on plant phenotyping and phenomics. Some of them are, Lemnatec (Germany), CSIRO (Australia), IBERS (U.K.), Universidad de Barcelona (Spain), the Julich Plant Phenomics Centre (German), and the James Hutton Institute (U.K.).

The establishment of LatPPN represented a big step forward toward the consolidation of a common mind-set in the field of plant phenotyping and phenomics across LAC. Clearly there are more opportunities than disadvantages, and each weakness needs to be addressed having in mind a regional approach.

## Conclusions and future work

Phenomics can complement the potential of new molecular/genotyping technologies, and together with agronomy and plant breeding efforts would be a real contribution to develop new strategies to help mitigate the impact of climate change in agriculture. There are major opportunities for phenomics in LAC, not only because it has been adopted in isolated initiatives, but also as worldwide development has focused mainly on grain breeding programs. LAC researchers have identified the need to collaborate to exploit the opportunities and gathered together to organize the Latin American Plant Phenomics Network (LatPPN). Currently, LatPPN has prioritized the work on several fronts to consolidate the network (e.g., grant application to CYTED and Procisur, LatPPN's second meeting in April 2016 (Balcarce, Argentina) and planning a second regional conference organized by EMBRAPA during 2017, drafting of LatPPN's survey, drafting of LatPPN's white paper, and construction of LatPPN's webpage). What follows next is the development of strategies leading to the sustainability of the network. We are aware of the work ahead of us and know that the collaboration within LatPPN members and with other networks will be crucial to build on the foundations laid.

## Author contributions

Both authors contributed equally.

### Conflict of interest statement

The authors declare that the research was conducted in the absence of any commercial or financial relationships that could be construed as a potential conflict of interest.
